# Effect of oxytocin added into sperm on artificial insemination in sheep

**DOI:** 10.5194/aab-66-61-2023

**Published:** 2023-02-07

**Authors:** Çağrı Kandemir

**Affiliations:** Department of Animal Science, Faculty of Agriculture, Ege University, 35000, Izmir, Türkiye

## Abstract

The use of additional oxytocin hormones in reproductive methods began in the 1970s in Europe. In recent studies, attempts have been made to achieve more successful pregnancies by adding oxytocin to semen rather than administering oxytocin exogenously to females. In light of this information, this study aimed to understand the effects of adding low-dose oxytocin hormone into ram semen on the pregnancy rate and reproductive efficiency of sheep. Semen was collected from five Chios rams. The ejaculates were pooled and Ovixcell^®^ was used as the diluent. The estrus was synchronized using an intravaginal sponge (Chrono-Gest^®^) from 122 Chios ewes. After the sponge was left in the vagina for 12 d, a 500 IU PMSG injection was made. In every sheep, intramuscular (IM) injections were inseminated by the intracervical method using an insemination gun 50 h after injection. Pregnancy results were obtained by ultrasound examination without practice. Lambing performance was recorded at delivery; 53 out of 122 Chios ewes were inseminated with oxytocin hormone content, and successful pregnancy was achieved in 90.56 % of them. In the insemination of the remaining 69 sheep, oxytocin was not added, and the rate of successful pregnancy was 76.81 % (p=0.046). The overall success rate based on the insemination results of all the sheep was determined to be 82.77 %. In an examination of the pregnancy rate, one of the indicators of reproductive performance, the difference between the groups is statistically significant (p≤0.05). Higher pregnancies were obtained in insemination with semen fluid containing oxytocin.

## 1 Introduction

Artificial insemination in sheep is an important reproductive technology. With the help of artificial insemination, male studs with superior genetics could help yield a much higher number of offspring (Robertson and Rendel, 1950). However, among the most important reasons why artificial insemination is not widely used in sheep today is the high cost of artificial insemination along with low pregnancy rates which could be considered unsuccessful (Olivera-Muzante et al., 2011; Lu et al., 2020). Achieving successful pregnancy rates would increase artificial insemination in sheep.

Many factors affect the impregnation of animals through artificial insemination. Among these factors – both physical and environmental – are species, breed, body condition score, number of lactations, milk yield, environmental temperature, uterine tone, insemination time, insemination operator, semen quality, reflux of semen, vaginal mucus and insemination depth (Cameron et al., 1986; Anzar et al., 2003; Kershaw et al., 2005). Optimizing the environmental effects could improve physical conditions. In order to ensure sufficient sperm accumulation in the uterus, the cervix needs to be passed at a certain level, which is one of the most important activities in achieving a successful pregnancy (Olivera-Muzante et al., 2020). Some factors may affect the progression of spermatozoa in the reproductive tract; these factors could be classified as mechanical (folds, crypts, cilia), cellular, physicochemical (vaginal secretion, cervical mucus) and hormonal factors (Çoyan, 2005; Ömür, 2014). Successful pregnancies cannot be measured solely by the success of forward motility of spermatozoa; the effects originating from the female are equally important. Several studies report the positive effect of insemination depth on success (Eppleston et al., 1994; Falchi et al., 2021; Gutierrez et al., 2022) – insomuch that being able to leave semen 1 cm forward affects the rate of a successful pregnancy by 7 %–12 % (Eppleston et al., 1994). The initial position of the semen released (pushing the semen forward or backward) and the condition of the vaginal mucus during the initial moment is also very important (Özmen and Cirit, 2020). The fluid in the vagina during estrus is called cervical mucus. Particularly in sheep, the amount, as well as the chemical and physical structure of vaginal mucus, affects the semen passing the cervix (Linford, 1974; Özmen and Cirit, 2020). In addition, the contractility of the female reproductive tract directly affects the result. The level of secreted prostaglandins and oxytocin, the hormone of the posterior pituitary lobe, determines the increase in the contractility of the reproductive canal. Increased uterine contractility facilitates the progression of spermatozoa (Hawk, 1983; Kunz et al., 1996; Gündoğan and Uçar, 2003; Suarez and Pacey, 2006; Miki and Clapham, 2013; Ömür, 2014; Akthara et al., 2021). Additionally, these contractions help remove the dead spermatozoa from the uterus (Gündoğan and Uçar, 2003). Senger (1999) has reported that prostaglandins (PGF2α and PGE1) in semen cause an increase in uterine tone. Furthermore, some special substances, such as phenylephrine and ergonovine, help transport spermatozoa (Hawk, 1983). However, hormones such as histamine, adrenaline and acetylcholine that are released due to stress have a negative impact and diminish the forward transport of semen (Holzmann, 2001).

Physiologically, the female sheep species' cervix structure is narrow, curved and sensitive (İleri et al., 2002). With folds much less than 1 cm, it is structured in a way to mislead the insemination gun (İleri et al., 2002; Lu et al., 2020). This structure makes it difficult to deliver semen inside (Deligiannis et. al, 2005; Kershaw et al., 2005). With the intracervical insemination method, it is observed that semen accumulates either in front of or slightly inside the cervix (Casali et al., 2017), which makes it difficult to meet with the oocyte. In order to address this problem, insemination could be done using laparoscopic methods. With the laparoscopic method, the aim is to enter surgically a place near the middle of the horn uterus, leaving the semen in the uterine cavity to ensure sufficient accumulation. This way, a direct transition is provided without ever touching the cervix (Kulaksız and Arı, 2016). Although laparoscopic insemination in sheep yields much more successful pregnancies compared to intracervical insemination, many factors affect both cost and success. It can be said that the laparoscopic insemination rate is lower due to the large number of equipment necessary, the high cost of the equipment, the need for surgical intervention and an experienced operator, and the stress on the animal (Kulaksız and Arı, 2016; Vallejo et al., 2019).

The intracervical method has been the most commonly used method for artificial insemination in sheep until now (Fernandez-Abella et al., 2003; Olivera-Muzante et al., 2011; Lopez-Perez et al., 2012; Galarza et al., 2020; Abadjieva et al., 2020; Madrigalia et al., 2021). Although the rate of successful pregnancies is relatively low in this method, it is preferred mainly because, compared to the laparoscopic method, it costs less, requires less experience and does not necessitate surgical intervention. Given the fact that the speed of spermatozoa in intracervical insemination is 80–100 µ s${}^{-1}$, they are expected to take about 2 h to arrive at the isthmus (İleri et al., 2002). However, it takes spermatozoa approximately 5 min to reach the isthmus, the fertilization site, which can be attributed to uterine contractility (Madill et al., 2000; Langendijk et al., 2003). It has been reported that in the intracervical method, insufficient uterine contractility and cervical dilatation are among the important factors that affect the successful pregnancy rate (Khalifa et al., 1992; Kiss and Mikkelsen, 2005; Prellwitz et al., 2019; Stellflug et al., 2001; Lu et al., 2020). Some studies have shown that exogenously administering oxytocin in amounts less than 400 IU increases uterine contractility (Sayre and Lewis, 1996, 1997; Stellflug et al., 2001). This shows that the exogenous use of oxytocin could lead to more contractions of the uterus, allowing the semen to move further. This and other similar applications could increase the success rate of intracervical insemination in sheep. However, when less than 400 IU of oxytocin is administered exogenously, uterine contractility begins to normalize after 15 to 60 min, depending on the physiological condition of the animal, which may reduce the rate of successful pregnancies (King et al., 1996; Sayre and Lewis, 1996, 1997; Stellflug et al., 2001; Viudes-de-Castro et al., 2009). When higher doses of oxytocin are administered exogenously, the structure of the endometrium will be disrupted, making embryo implantation difficult (Jenkin, 1992; King et al., 1996; Stellfug et al., 2001; Beretsos et al., 2006). Further research is necessary to determine the appropriate dose required to achieve uterine contractility (Heppelmann et al., 2018).

The use of additional oxytocin hormones in reproductive methods began in the 1970s in Europe (Duzinski et al., 2013). In recent studies, attempts have been made to achieve more successful pregnancies by adding oxytocin to semen rather than administering oxytocin exogenously to females (Pena et al., 1998; Langendijk et al., 2003; Duzinski et al., 2013; Okazaki et al., 2014; Kandemir et al., 2017; Manjarin, et al., 2019; Lu et al., 2020). While these are being done, the effects of the antioxidant property of oxytocin should be investigated. The antioxidant effect of oxytocin should be examined after semen is stored for long periods or after freezing and thawing. Examination of these effects in ram semen will guide future research. However, this research aimed to understand the effects of adding low-dose oxytocin hormone into ram semen on the pregnancy rate and reproductive efficiency of sheep.

## 2 Materials and methods

### 2.1 Experimental animals, location and management

This study was conducted in October, during the breeding season of sheep, and lamb births took place in March. The research was achieved in the city of Izmir, located between 39${}^{\circ }$15${}^{\prime }$–37${}^{\circ }$45${}^{\prime }$ north latitudes and 28${}^{\circ }$20${}^{\prime }$–26${}^{\circ }$15${}^{\prime }$ east longitudes, with an average temperature of 10–12 ${}^{\circ }$C. In the study, 122 Chios ewes were selected from a herd of 280 ewes; the sheep in the sample were clinically determined to have no reproductive problems; they have birthed twice, and their live weights ranged from 57 to 70 kg. The rams included in the study were five Chios rams, over 3 years old with no health and reproductive problems, weighing about 94.65 ± 1.78 kg. Calculating 1.6 times the daily energy expenditure for sheep and 1.2 times for rams, rations were prepared by NRC (2007; Table 1). Permission from the ethics committee regarding the animal experiments was received for this study (Republic of Türkiye Ministry of Agriculture and Forestry). All procedures performed in this study followed the ethical principles of the Declaration of Helsinki.

**Table 1:** Analysis results of feed given to ewes and rams. DM: dry matter. CP: crude protein.

Alfalfa (%)		Wheat straw (%)		Corn silage (%)		Concentrated feed (%)		Barley (%)	
DM	CP	DM	CP	DM	CP	DM	CP	DM	CP
89.55	17.98	90.35	10.28	91.02	10.25	90.33	17.78	90.21	10.55

### 2.2 Semen preparation

Semen was collected from five Chios rams with the aid of an artificial vagina (Minitube^®^, Germany). Semen characteristics include a volume of 1.1–2.1 mL semen with semen density $>3\times 10^{9}$, and spermatozoa motility >70 % was pooled by mixing. Ovixcell^®^-IMV Technologies (France) were used to increasing the volume of semen. The total amount of semen was divided into two groups: experimental and control. While nothing was added to the control group, 0.5 U of Oxytocin (Vetaş^®^ Brand, Türkiye) per millilitre of Ovixcell^®^ was added to the experimental group 15 min before the insemination (Lu et al., 2020). The semen was kept at 30 ${}^{\circ }$C until the insemination procedure.

### 2.3 Oestrous synchronization

To ensure oestrous synchronization, intravaginal sponges (Chrono-Gest^®^, Germany) containing hormones were inserted into all of the sheep used in the study. The sponges remained in situ for 12 d, and immediately after sponge removal, 500 IU PMSG (Chrono-Gest^®^, Germany) IM was administered.

### 2.4 Artificial insemination

Intracervical artificial insemination of sheep was done between 50–55 h after sponge removal. Speculum and source of light were used during insemination. The semen, containing $125\times 10^{6}$ spermatozoon, was applied once, using 0.25 mL straws to allow penetration as far into the cervix as possible. In order to avoid any time difference, the insemination sequence was performed alternatively on control and experimental groups. A total of 122 ewes, 69 in the control group and 53 in the experimental group, were inseminated by the same technician.

During the evaluation of the factors affecting the insemination results, the groups were not arranged randomly but formed according to the status encountered during insemination; i.e. if a small amount of fluid was encountered in the vagina during insemination, the group was classified as little; if there was a large amount of fluid, it was classified as large; and those that had a moderate amount of fluid were included in the moderate cervical mucus fluid group. Similarly, groups were classified according to the insemination depth results obtained. Inseminations were carried out in such a way that they could enter through the cervix with an insemination gun as much as possible; the classification of the groups was made as intracervix-2 for those with an entry of approximately 2 cm, intracervix-1 for those with an entry of approximately 1 cm, and as anterior cervix for those with no cervix entry. An insemination pipette with a different colour and with the dimensions on its tip was used when entering the cervix. This tip is 1 cm long and is in shades of blue. This straw allowed us to measure inflows in sheep with a lot of cervical mucus and a bit of manual pressure under the abdomen. Another feature investigated was the reflux of semen behaviour in the uterus. If absorption behaviour occurred after the semen was released immediately after entry with the insemination gun, the group was defined as non-rebounding; if no absorption behaviour was observed, it was defined as rebounding.

### 2.5 Diagnosis and lambing

A pregnancy examination was performed 46 d after artificial insemination. Pregnancy was diagnosed by ultrasonography. B-mod real-time ultrasound with a 3.5 MHz probe (Mindray DP-50 Vet) was used. Those with at least one fetus were defined as pregnant. The features examined in the research, birth weight and gestational age were recorded after delivery.

pregnancy rate = number of pregnant sheep/number of inseminated sheep,
multiple birth rate = number of twin born lambs/total number of lambs,
lambing rate = number of born lambs/number of pregnant sheep,
number of lambs at birth = number of single or twin lambs/total number of lambs,
litter size = number of lambs born in the group/total number of lambs,
female lamb rate = number of female lambs born/total number of lambs,
male lamb rate = number of male lambs born/total number of lambs

### 2.6 Statistical analysis

Data are presented as mean, and p≤0.05 is considered significant for the probability value. Student's t test was used to evaluate the data at birth and lamb birth weights. Chi-square test was done to determine the difference between the groups of other features measured. All analyses were conducted using the IBM^®^ SPSS Statistics 20 statistical package program.

## 3 Results

The study investigated the effects of semen diluent (containing or not containing oxytocin) on reproductive performance. Pregnancy rate, multiple birth rate, lambing rate, litter size, female lambing rate, male lambing rate, and birth weights of lambs were measured in order to evaluate the reproductive performance. Table 2 illustrates the effects of semen fluids containing oxytocin and no oxytocin on reproductive performance.

**Table 2:** Reproductive performance values of sperm fluids containing oxytocin and no oxytocin.

Reproductive characteristics	No oxytocin	Oxytocin	Total	p value	
Pregnancy rate (%)	76.81 (53/69)${}^{\text{b}}$	90.56 (48/53)${}^{\text{a}}$	82.77 (101/122)	0.046	
Multiple birth rates (%)	44.64 (25/56)	38.29 (18/47)	41.74 (43/103)	0.478	
Lambing rate (%)	100.00 (103/103)	100.00 (103/103)	100.00 (103/103)	1.000	
Number of lambs					
– single (%)	51.67 (31/60)	48.33 (29/60)	58.25 (60/103)	0.424	
– twin (%)	58.14 (25/43)	41.86 (18/43)	41.74 (43/103)	0.424	
Litter size (%)	55.44 (56/101)	46.53 (47/101)	1.02 (103/101)	1.000	
Female lamb rate (%)	48.00 (24/50)	52.00 (26/50)	1.00 (50/50)	0.842	
Male lamb rate (%)	47.17 (25/53)	52.83 (28/53)	1.00 (53/53)	0.842	
Birth weight					
– single (%)	4.52 ± 0.36	4.46 ± 0.44	4.49 ± 0.39	0.344	
– twin (%)	3.89 ± 0.52	3.77 ± 0.29	3.80 ± 0.34	0.745	
Period of gestation	150.6 ± 1.16	150.1 ± 1.82	150.3 ± 1.57	0.960	

${}^{\text{a, b}}$ The difference between values with different letters in the same row is significant at the p value.

In the examination of the pregnancy rate, one of the indicators of reproductive performance, the difference between the groups is statistically significant (p=0.046; p≤0.05). Higher pregnancies were obtained in insemination with semen fluid containing oxytocin. On the other hand, other reproductive performances examined in the study were not found to be statistically significant (p>0.05).

The results of the semen rebounding behaviour depend on the amount of vaginal mucus, insemination depth and uterine contractility, which is thought to affect the insemination results, as given in Table 3. The amount of vaginal mucus during insemination was found to be statistically significant in achieving successful pregnancy (p=0.000; p≤0.05). The increase in a vaginal fluid is seen to reduce the pregnancy rate. On the other hand, insemination depth and reflux of semen behaviour were not found to be statistically significant (p>0.05). Although the insemination depth is not statistically significant, it is observed that the success rate is higher as entry into the cervix is deeper. If the number of animals is high in commercial establishments, this ratio could lead to a financially significant amount.

**Table 3:** Values of factors affecting insemination results.

	Non-pregnant	Pregnant	p value
Vaginal mucus			
Few	12.24 (12/98)	87.76 (86/98)${}^{\text{a}}$	
Middle	35.71 (5/14)	64.29 (14/19)${}^{\text{b}}$	<0.001
Numerous	80.00 (4/5)	20.00 (1/5)${}^{\text{c}}$	
Insemination depth			
Anterior of the cervix	25.00 (2/8)	75.00 (6/8)	
Intracervix-1	16.47 (14/85)	83.53 (71/85)	0.830
Intracervix-2	17.24 (5/29)	82.76 (24/29)	
Reflux of semen			
Backfire	17.64 (12/68)	82.36 (56/68)	0.887
Recoilless	16.67 (9/54)	83.33 (45/54)	

${}^{\text{a, b, c}}$ The difference between values with different letters in the same row is significant at the p value.

## 4 Discussion

In line with the aim of the study, it was determined that in Chios sheep whose heat was synchronized with exogenous reproductive hormones, the amount of oxytocin hormone added to the semen diluter had a high positive effect on success even with monochronic insemination. A total of 53 out of 122 Chios ewes were inseminated with oxytocin hormone content, and successful pregnancy was achieved in 90.56 % of them. Several studies report that the oxytocin hormone has been used to achieve successful pregnancy from the past to the present. In these studies, some researchers added the hormone into the semen, while others administered it to the animal intramuscularly (IM). Similarly to this study but in different animal species, added to semen, in their study on 1373 pigs, Hüln et al. (1977) reported that the results of insemination with the hormone oxytocin added to the semen were superior to those of the control group. Fuchs et al. (1989) reported that the use of oxytocin in cattle semen increased uterine contractions and the forward transport of spermatozoa. In their study conducted during the summer months, Pena et al. (1998) reported that a 77.02 % pregnancy rate obtained with oxytocin hormone added into pig semen, compared to the 54.39 % pregnancy rate obtained in the control group, was statistically significant. In their 2-year study on pigs, Duzinki et al. (2014) reported that based on 10 486 insemination results, semen with added oxytocin significantly increased pregnancy rates in all seasons. Okazaki et al. (2014) performed insemination with both fresh and frozen semen with the hormone oxytocin added to the porcine semen diluter. They reported results of 87.50 % in the oxytocin group, 70.50 % in the control group in the inseminations performed with fresh semen; in those performed with frozen and thawed semen, the results were 89.80 % in the oxytocin group, and 75.00 % in the control group. In their study of 800 inseminations in pigs 2 years in a row, Manjarin et al. (2019) reported positive results of oxytocin addition into the semen. This study and other research show that the oxytocin hormone added to the semen diluter helps transport sperm to the oviduct in the uterus. Similarly to this study, in the same animal species, Lu et al. (2020) used a diluter containing oxytocin in the insemination of 199 Kazakh sheep with oestrous synchronization. The pregnancy test results of the group receiving diluent with oxytocin were 85.50 %, whereas the result of 68.80 % was obtained in the control group. Researchers reported that the difference between these groups was statistically significant. These studies' results are congruent with those obtained in this study. The oxytocin hormone added to the semen may increase contractions as it progresses in the uterus, which would help the spermatozoa to move forward faster. As such, more spermatozoa will reach the female reproductive cell, possibly increasing the rate of successful pregnancies.

Many factors affect the achievement of successful pregnancy in artificial insemination in sheep. Factors such as semen density, diluent, insemination time and semen storage are among the most important. In studies conducted on sheep at different times, it was reported that pregnancy rates between 11 % and 55 % were obtained in insemination with fresh semen. Olivera et al. (2005) reported that in inseminations performed with spermatozoon density of $120\times 10^{6}$ at 24th and 48th hours, prepared with diluents containing tris and skim milk, kept in +5 ${}^{\circ }$C, they achieved pregnancy rates of 19 %, 22 %, 49 % and 47 % respectively. Menchaca et al. (2005) reported that in inseminations made with $200\times 10^{6}$ spermatozoa density at the 24th and 48th hours, prepared with diluents containing tris, glycerol, citric acid and egg yolk, they achieved pregnancy rates of 54 %, 43 % and 35 %, respectively. It is seen that the best pregnancy results are obtained with the semen waiting for the shortest time. In these research, semen density and holding temperatures similar to this study were used. However, the semen holding time is much shorter. In the study of Olivera et al. (2005) and Menchaca et al. (2005), it can be said that the pregnancy rate is low because the semen retention time is longer and different diluents are used. During the preparation of semen, the effect of the extender can directly affect the result. During this research, Ovixcell commercial diluter was used as a diluent, unlike Naim et al. (2009), who reported that in inseminations prepared with OviPro commercial diluter, with spermatozoa densities of $150\times 10^{6}$ and $300\times 10^{6}$ kept at +5${}^{\circ }$ and performed within 24 h; they obtained 11 % and 29 % pregnancy rates respectively. Using different diluents Cueto and Gibson (2010) reported that a 55 % pregnancy rate was achieved in inseminations done with $150\times 10^{6}$ spermatozoa density between 6–8 h, prepared with a skim milk diluent kept at +15${}^{\circ }$. The effect composition of the diluents was directly effective in the low pregnancy outcomes, according to this research. It could be seen that the pregnancy results obtained in the present study are better than those previously reported. The reason for this could be attributed to the fact that the ideal factors affecting the success of artificial insemination were selected. Studies on sheep insemination continue to be carried out today. The majority of these studies are similar to this study, with intracervical insemination using fresh semen. Pesan et al. (2021) reported that they achieved a 63.90 % pregnancy rate in inseminating Zwartbles sheep. Madrigalia et al. (2021) reported that they obtained an average rate of 40.80 % in the insemination of Assaf and Awassi breeds in different businesses in Italy. Asaduzzaman et al. (2021) stated that the pregnancy rate obtained in their study was 79.16 %. Kutluca Korkmaz and Yaprak (2022) reported that in their research, the average pregnancy rate in Red Karaman sheep was 74.00 %. The 82.77 % pregnancy rate obtained in the study is congruent with existing research. It is thought that obtaining different results may stem from many factors that affect the success of insemination. Chios Sheep is among valuable sheep breeds in the world due to the high number of lambs at birth. Many studies that describe the reproductive performance of the Chios breed have been conducted. Akçapinar et al. (2005) reported the results of Chios and Karayaka crossbreeds as 32 % and 52 % in two different experimental groups. Abd-allah et al. (2011) reported that they obtained 30 % multiple births in a study involving 111 Chios sheep under Egyptian conditions. In their study on Chios sheep, Linda et al. (2020) reported a multiple birth rate of 90 % among 2315 sheep. Although the rate of 41.74 % obtained in the study appears to be somewhat low, it is within the values reported for the Chios breed. The low multiple birth rate obtained in this study may be associated with the genetics of the inseminated population.

The study was conducted using the intra-cervical method with a speculum and light source. The vaginal mucus is seen before artificial insemination was classified based on the amount. In those sheep with a high amount of vaginal mucus, the pregnancy rate was determined as 20.00 %. However, in sheep with moderate and low levels of vaginal mucus, the success rate was determined as 64.29 % and 87.76 % respectively. Many studies have examined the ideal time for insemination in order to achieve successful pregnancies. The ideal time for insemination time is associated with the general condition of the vaginal mucus (Murtaza et al., 2020). Mahmoudzadeh et al. (2001) reported that the amount of vaginal mucus does not affect the success of insemination in cattle. However, Abril-Parreno et al. (2021) reported that the amount of cervical mucus in different sheep varies according to their breed and that the amount alone cannot be determinative. The present study finds that a high amount of vaginal mucus in Chios sheep negatively affects insemination success. The high amount is thought to increase the rate of ejection from the vulva due to the mass movement of the semen released into the vaginal mucus. Hence, it is thought that the rate of successful pregnancies is higher in sheep with a low or moderate amount of vaginal mucus.

## 5 Conclusions

As per the findings obtained in the research, oxytocin hormone added to ram semen diluter significantly increases the rate of successful pregnancy in sheep. Additionally, it was found that the amount of vaginal mucus during insemination adversely affects the success of insemination. In order to achieve a successful pregnancy, it will be beneficial to remove excess vaginal mucus with the help of an aspirator during insemination in sheep. In light of all these findings, it would be beneficial to conduct studies involving insemination in higher numbers in different breeds to better understand the effect of the oxytocin hormone on pregnancy.

## Data Availability

The data used and analyzed during this study are available from the corresponding author upon reasonable request.
