# Corrigendum to “Effect of oxytocin added into sperm on artificial insemination in sheep” published in Arch. Anim. Breed., 66, 61–69, 2023

**DOI:** 10.5194/aab-69-215-2026

**Published:** 2026-03-31

**Authors:** Çağrı Kandemir

**Affiliations:** 1 Department of Animal Science, Faculty of Agriculture, Ege University, 35000, Izmir, Türkiye

There is an error in Tables 2 and 3 of the published paper. The ratios based on the number of lambs have been updated. The corrected versions of Tables 2 and 3 can be found below.

**Table 2:** Reproductive performance values of sperm fluids containing oxytocin and no oxytocin.

Reproductive characteristics	No Oxytocin	Oxytocin	Total	p value
Pregnancy rate (%)	76.81 (53/69)^b^	90.56 (48/53)^a^	82.77 (101/122)	0.046
Multiple birth rates (%)	47.17 (25/53)	37.50 (18/48)	42.57 (43/101)	0.416
Lambing rate (%)	100.00 (53/53)	100.00 (48/48)	100.00 (101/101)	1.000
Number of lambs				
Single (%)	35.90 (28/78)	45.45 (30/66)	40.28 (58/144)	0.368
Twin (%)	64.10 (50/78)	54.55 (36/66)	59.72 (86/144)	0.368
Litter size (%)	1.47 (78/53)	1.38 (66/48)	1.43(144/101)	0.426
Female lamb rate (%)	48.71 (38/78)	48.48 (32/66)	48.61 (70/144)	0.482
Male lamb rate (%)	51.28 (40/78)	51.52 (34/66)	51.39 (74/144)	0.482
Birth weight				
Single (%)	4.49 ± 0.34	4.45 ± 0.48	4.47 ± 0.38	0.462
Twin (%)	3.78 ± 0.48	3.74 ± 0.34	3.76 ± 0.29	0.462
Period of gestation	150.6 ± 1.16	150.1 ± 1.82	150.3 ± 1.57	0.960

^a,b^ The difference between values with different letters in the same row is significant at the p value.

**Table 3:** Values of factors affecting insemination results.

	Non-pregnant	Pregnant	p value
Vaginal mucus			
Few	12.24 (12/98)	87.76 (86/98)^a^	< 0.001
Middle	26.32 (5/19)	73.68 (14/19)^b^	
Numerous	80.00 (4/5)	20.00 (1/5)^c^	
Insemination depth			
Anterior of the cervix	25.00 (2/8)	75.00 (6/8)	0.830
Intracervix-1	16.47 (14/85)	83.53 (71/85)	
Intracervix-2	17.24 (5/29)	82.76 (24/29)	
Reflux of semen			
Backfire	17.64 (12/68)	82.36 (56/68)	0.887
Recoilless	16.67 (9/54)	83.33 (45/54)	

^a,b,c^ The difference between values with different letters in the same row is significant at the p value.

